# Geographical patterns in blood lead in relation to industrial emissions and traffic in Swedish children, 1978–2007

**DOI:** 10.1186/1471-2458-9-225

**Published:** 2009-07-10

**Authors:** Emilie Stroh, Thomas Lundh, Anna Oudin, Staffan Skerfving, Ulf Strömberg

**Affiliations:** 1 Department of Laboratory Medicine, Lund University, Lund, Sweden

## Abstract

**Background:**

Blood lead concentrations (B-Pb) were measured in 3 879 Swedish school children during the period 1978–2007. The objective was to study the effect of the proximity to lead sources based on the children's home and school location.

**Methods:**

The children's home address and school location were geocoded and their proximity to a lead smelter and major roads was calculated using geographical information system (GIS) software. All the statistical analyses were carried out using means of generalized log-linear modelling, with natural-logarithm-transformed B-Pb, adjusted for sex, school year, lead-exposing hobby, country of birth and, in the periods 1988–1994 and 1995–2007, parents' smoking habits.

**Results:**

The GIS analysis revealed that although the emission from the smelter and children's B-Pb levels had decreased considerably since 1978, proximity to the lead smelter continued to affect levels of B-Pb, even in recent years (geometric mean: near smelter: 22.90 μg/l; far from smelter 19.75 μg/l; p = 0.001). The analysis also revealed that proximity to major roads noticeably affected the children's B-Pb levels during the period 1978–1987 (geometric mean near major roads: 44.26 μg/l; far from roads: 38.32 μg/l; p = 0.056), due to the considerable amount of lead in petrol. This effect was, however, not visible after 1987 due to prohibition of lead in petrol.

**Conclusion:**

The results show that proximity to the lead smelter still has an impact on the children's B-Pb levels. This is alarming since it could imply that living or working in the vicinity of a former lead source could pose a threat years after reduction of the emission. The analysis also revealed that urban children exposed to lead from traffic were only affected during the early period, when there were considerable amounts of lead in petrol, and that the prohibition of lead in petrol in later years led to reduced levels of lead in the blood of urban children.

## Background

**Figure 1:**
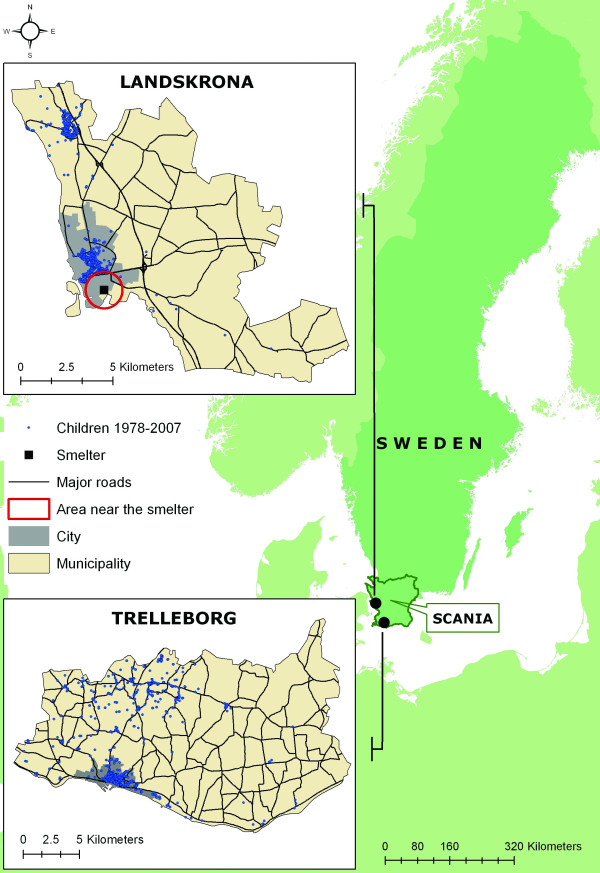
**Study location**. Map showing the location of the county of Scania, and the municipalities of Landskrona and Trelleborg. The major roads in the municipalities, the location of the lead smelter in Landskrona and the location of the participating children's residences, as well as the area defined as "near the smelter", and city areas from previous studies.

Lead exposure is a major health problem, especially in children [1]. Since 1978, annual measurements have been made of the blood lead concentration (B-Pb) in children living in the municipalities of Landskrona and Trelleborg, both located on the coast in southern Sweden, separated by a distance of 70–80 km (Figure 1). We have previously reported results from this unique time series of B-Pb measurements in children [2-4]. In 1978 the geometric mean B-Pb level was 60 μg/l, whereas in 2007 it was only 13 μg/l, indicating a dramatic decrease in B-Pb during the period 1978–1994 [4].

Lead exposure may arise from several sources. A major source to lead exposure in many parts of the world is leaded house paint. In Sweden the use of leaded house paint was prohibited in 1926 hence, lead-paint is not an exposure source here. An important exposure factor might instead be caused by, in some areas, lead emissions from industries. However, lead from petrol has been a major source of exposure in most countries. Petrol sold in Sweden after 1994 has not contained lead (National Swedish Environment Protection Agency; Swedish Petroleum Institute), and the decrease in B-Pb levels in children in Landskrona and Trelleborg reflects the gradual reduction of the use of leaded petrol [2-4]. The rate of decrease of B-Pb observed in children during the period 1978–1994, when leaded petrol was phased out, was found to be similar to that during the period 1995–2007, when only unleaded petrol was available, viz. close to 5% per year [3]. Hence, the B-Pb levels in children continued to decline noticeably more than a decade after leaded petrol had been phased out in Sweden.

A secondary lead smelter, located about 1 km from the town centre, has been in operation in Landskrona since 1944, while there is no lead-emitting industry in Trelleborg. The children studied were previously crudely grouped according to their residential area; in Landskrona: those who lived near the smelter (i.e. the industrial area), those living in urban areas, and those in rural areas, and in Trelleborg: children living in urban and rural areas (Figure 1). A "near-smelter" effect, reflecting the impact of industrial emissions on children's B-Pb levels, has been observed throughout the duration of the study [2-4]. However, there are several concerns about this crude geographical grouping of the children, namely: (i) the exact residential location was not used, (ii) the location of the children's school was not taken into consideration, and (iii) the distinction between urban and rural areas does not necessarily reflect the degree of exposure arising from traffic, as major roads were not taken into consideration. To resolve these problems we have applied spatial analysis through the use of GIS in this study by linking each child's home and school address to their geographical coordinates and thus enabled us to analyse the geographical interactions between the children's daily dwelling and their proximity to the smelter and major roads in their vicinity with high accuracy.

Similar studies have used GIS successfully to analyse the importance of proximity to lead sources and elevated blood lead levels [5-8] or to classify risk areas and groups for further investigation and action plans [9-12]. Our study differs from these due to the length of the study (1978–2007) that covers both the period when lead was a substantial substance in petrol, the phase out and the lead free period. This analysis also applies spatial analyses to evaluate the importance of children's daily proximity to the lead smelter by analysing their home and school location as well as closeness to a major road nearby their home. This approach enables us to analyse both spatial relationships between the children's dwelling and the smelter/closest major road with high accuracy as well as guesstimate the impact of both traffic and the emissions from the lead smelter on the children's blood lead levels.

This article focuses on evaluating the geographical patterns of lead exposure among children, with special reference to industrial emissions and traffic over time.

## Methods

### Study areas

The municipalities of Landskrona and Trelleborg are situated in the southernmost county of Sweden, Scania (Figure 1). In 2007, the population in each of the two municipalities constituted approximately 3% (40 000) of the total population in Scania (1 198 000). The geographical distribution of the populations of the two municipalities are similar, with about 80–90% living in urban areas. An area of specific interest in Landskrona is the industrial area situated in the southernmost part of the city, where a lead smelter is located (Figure 1). There have been no industrial lead emissions in Trelleborg, and it is therefore considered to be a suitable control area, due to the similar constitution of the population to that in Landskrona.

### Study population

Parents have been invited to allow their children to participate in the study since 1978. In 1978, the participation of children aged between 10 and 17 years was encouraged; later, the age interval was changed to 8–10 years (in 1986 preschool children from 3 years of age also took part, n = 77). Generally, children who lived in Landskrona and Trelleborg, respectively, were invited to participate every second year). Children attending major schools in the two municipalities were requested to participate in the study. The schools were selected according to their location in order that 1/3 of them would be situated close to the smelter, 1/3 in the urban area/vicinity of the urban area and 1/3 further away. The parents to the children (primarily) attending 1st to 3rd grade in these schools received an informative letter regarding the study and the children who wished to participate and had informed consent by their parents was then interviewed and had a blood sampling conducted. The overall participation rate was about 60% (across all schools and school years during the whole study period) which, regarding to the low age of the children and the fact that they had to leave a blood sample is relatively high.

During the period 1978–2007, 4218 individual blood samples were collected from 3713 children and analysed with regard to B-Pb level (more than one sample was obtained from 481 children in different years, and three or more samples were obtained from 24 children). In addition, all the participating children were questioned regarding their hobbies (to ascertain whether they involved potential lead exposure, e.g., shooting air guns, making lead soldiers, etc.) During the period 1988–2007, their parents' smoking habits were recorded. The preschool/school attended by each child was recorded, together with their school year (based on their year of birth), and country of birth (data taken from the Regional Population Register).

Children entered the study only after informed consent was obtained from their parents. The study was approved by the Ethics Committee of the Medical Faculty, Lund University, Sweden.

### Blood lead analysis

The methods for blood lead analysis have been described in our previous reports [2-4]. Accuracy, checked by control blood samples, has been satisfactory.

### Geographical analysis

The children participating in the study from 1983 were geocoded by linking their personal identification number to the centre coordinates of their home (as listed in the Regional Population Register). Since the Regional Population Register lacked housing information for previous years, the residential locations of the children participating in the study from 1978–1982 were obtained by coding their home address taken from old school records, and then matching these data with a geocoded address database. In total, we obtained the relevant geographical coordinates for 3571 children. The remaining 142 children could not be geocoded due to incomplete information, and were therefore not included in the analysis. As multiple B-Pb measurements had been made on several children, 3917 B-Pb measurements were included in this study.

Geocoded data for major roads (i.e. motorways and main routes) covering both municipalities, during the whole study period (1978–2007) were obtained from the Swedish Road Administration. The locations of the children's schools, and the lead smelter in Landskrona, were geocoded by matching their addresses with the geocoded address database. The Euclidian distance between the location of their home and the closest major road in the municipality was calculated for each child. Two additional proximity calculations were performed for children living in Landskrona, based on the distance between the lead smelter, and their home and school.

A time-weighted distance was constructed for each child who lived in Landskrona, based on the assumption that the children spent approximately 20% (4 hours per day) of each working day at school, and the remaining 80% at home, i.e., 0.8α + 0.2β, where α denotes the distance between the child's home and the lead smelter, and β denotes the distance between the location of the child's school and the lead smelter. During the early period, 1978–1987, information on school attendance by children participating in 1978 was not collected (in total 500 children). The geometric mean of the B-Pb levels of these children was 59.7 μg/l (range: 18.0–249.6 μg/l; SD: 1.4 μg/l). Of these, the homes of 228 children were 0.9–2 km from the smelter, 146 were 2–3 km from the smelter, and the remaining 126 children lived further than 3 km from the smelter.

### Statistical methods

The geographical patterns of lead exposure among the children were analysed by means of generalized log-linear modelling, with natural-logarithm-transformed B-Pb level as the dependent variable (geometric mean of B-Pb), and the following fixed-effect factors: sex, school year (preschool, 1st, 2nd, 3rd, > 3rd), potentially lead-exposing hobby (yes, no), country of birth (Nordic countries, other countries), parents' smoking habits (at least one smoking parent, no smoking parent), and a geographical variable (see below). Separate analyses were carried out for three different periods: 1978–1987 (high levels of lead in petrol; no data on parents' smoking habits available), 1988–1994 (decreasing use of leaded petrol), 1995–2007 (unleaded petrol only). In order to study the geographical patterns of B-Pb levels within each period, geometric means of B-Pb levels, with 95% confidence intervals (CIs), were estimated from the fitted models (yielding average levels adjusted for possible confounding effects of the covariates). All the statistical analyses were carried out using geometric mean of B-Pb levels adjusted for sex, school year, lead-exposing hobby, country of birth and, in the periods 1988–1994 and 1995–2007, parents' smoking habits. A few children contributed with multiple B-Pb measurements across different school years (see above). We also carried out repeated measure modelling, taking into account reasonable correlation structures between the residuals for the repeated measurements. The results based on the repeated measurement models were not notably different (geometrical mean did not differ more than 7%; and the standard errors did not differ more than 5% except for Trelleborg during the period 1995–2007 when the standard error differed up to 18%) from the results based on the corresponding models assuming uncorrelated residuals over all measurements (i.e., all data treated as independent measurements); we present the results based on the latter models.

The geographical patterns of B-Pb levels with reference to traffic were described by grouping the children into categories based on the proximity of their homes to major roads (0–50, 50–100, 100–200 and >200 m). The geographical patterns with reference to industrial emission were described by grouping the Landskrona children into categories based on the distances from the lead smelter (0.9–2, 2–3 and >3 km), to their home and school, including weighting for the time spent in school and at home. In order to construct an informative map of B-Pb levels in Landskrona, the children were also grouped into five different categories based on their B-Pb level (0–25, 26–50, 51–75, 76–100 and >100 μg/l). For each of these B-Pb categories, the geographical mean was assessed by calculating the average x and y coordinates based on the home locations of the children.

SPSS for Windows, release 13 [13] was used for the statistical analyses.

## Results

Table 1 provides a summary of the data analysed.

**Table 1:** Background information

	**Landskrona**			**Trelleborg**		
	**1978–1987**	**1988–1994**	**1995–2007**	**1978–1987**	**1988–1994**	**1995–2007**
**N**	979	657	890	652	238	501

**Geometric mean B-Pb (μg/l)**	49.7	30.9	18.4	46.5	28.2	18.2
Range	14.0 – 249.6	11.0 – 122.7	5.7 – 79.8	14.0 – 162.4	10.0 – 68.0	6.1 – 62.0
>50 μg/l, N (%)	488 (49.8)	57 (8.7)	15 (1.7)	271 (41.6)	11 (4.6)	2 (0.4)

**Sex, N (%)**						

Girl	467 (47.7)	355 (54.0)	431 (48.4)	344 (52.8)	115 (48.3)	259 (51.7)
Boy	512 (52.3)	302 (46.0)	459 (51.6)	308 (47.2)	123 (51.7)	242 (48.3)

**School year, N (%)** *B-Pb range (μg/l)*						

Preschool (4–7 years)	77 (7.9) *21 – 84*	0 (0.0) ---	0 (0.0) ---	0 (0.0) ---	0 (0.0) ---	0 (0.0) ---
1^st ^(8 years)	42 (4.3) *24 – 88*	140 (21.3) *12 – 73*	111 (12.5) *6 – 55*	146 (22.4) *14 – 162*	29 (12.2) *16 – 68*	80 (16.0) *7 – 40*
2^nd ^(9 years)	164 (16.8) *14 – 129*	303 (46.1) *11 – 122*	352 (39.6) *6 – 72*	151 (23.2) *16 – 115*	93 (39.1) *10 – 67*	175 (34.9) *6 – 49*
3^rd ^(10 years)	150 (15.3) *15 – 102*	214 (32.6) *13 – 68*	410 (46.1) *6 – 80*	137 (21.0) *15 – 124*	116 (48.7) *12 – 64*	246 (49.1) *8 – 62*
Intermediate stage and senior levels (>10 years of age)	546 (55.8) *16 – 250*	0 (0.0) ---	17 (1.9) *12 – 68*	218 (33.4) *19 – 134*	0 (0.0) ---	0 (0.0) ---

**Smoking parents, N (%)**						

Yes (mother/father or both)	6 (0.6)	367 (55.9)	334 (37.5)	66 (10.1)	140 (58.8)	240 (47.9)
No	2 (0.2)	280 (42.6)	376 (42.2)	49 (7.5)	98 (41.2)	261 (52.1)
Missing	971 (99.2)	10 (1.5)	180 (20.2)	537 (82.4)	0 (0.0)	0 (0.0)

**Potentially Pb-exposing hobby, N (%)**						

Yes	92 (9.4)	126 (19.2)	128 (14.4)	81 (12.4)	57 (23.9)	66 (13.2)
No	887 (90.6)	531 (80.8)	762 (85.6)	571 (87.6)	181 (76.1)	435 (86.8)

**Birth country, N (%)**						

Nordic countries	936 (95.6)	622 (94.7)	809 (90.9)	623 (95.6)	231 (97.1)	461 (92.0)
All other countries	31 (3.2)	35 (5.3)	79 (8.9)	21 (3.2)	7 (2.9)	40 (8.0)
Missing	12 (1.2)	0 (0.0)	2 (0.2)	8 (1.2)	0 (0.0)	0 (0.0)

Background information for the 3 917 measurements on blood-lead concentrations (B-Pb) in Swedish children, carried out during the period 1978–2007

**Figure 2:**
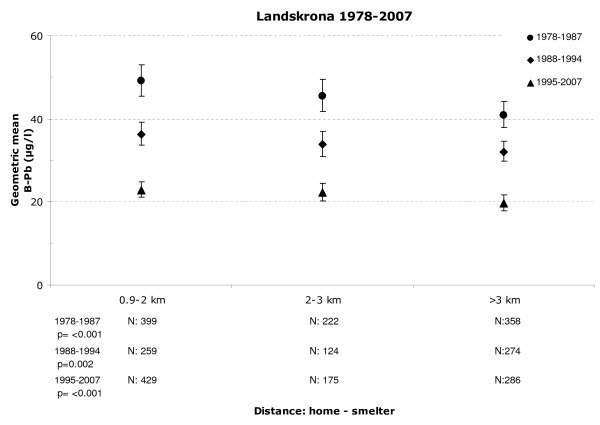
**Geometric mean of blood lead based on the distance between the home and the lead smelter**. Geometric mean of blood lead levels of children in Landskrona as a function of the distance between their home and the lead smelter. Whisker represents the 95% confidence interval.

In Landskrona, the geometric mean of the B-Pb levels changed significantly according to the proximity to the lead smelter (Figure 2, numbers and p-values are presented in the figures). This pattern was especially evident during the earlier period, 1978–1987, but a statistically significant effect of the proximity to the smelter was also evident in the later periods.

**Figure 3:**
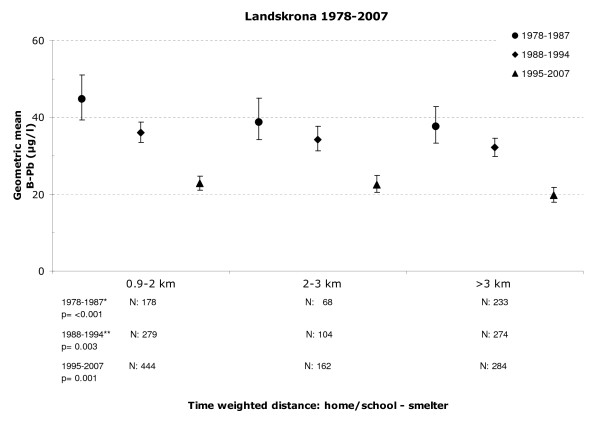
**Geometric mean of blood lead based on the time-weighted measure of the home/school and the lead smelter**. Geometric mean of blood lead levels of children in Landskrona as a function of the distance between the time-weighted measure of their home and school and the location of the smelter. Whisker represents the 95% confidence interval.

The significant association between proximity to the lead smelter and the children's B-Pb levels persisted when the distance was adjusted for time spent in school, i.e. the time-weighted distance (Figure 3).

**Figure 4:**
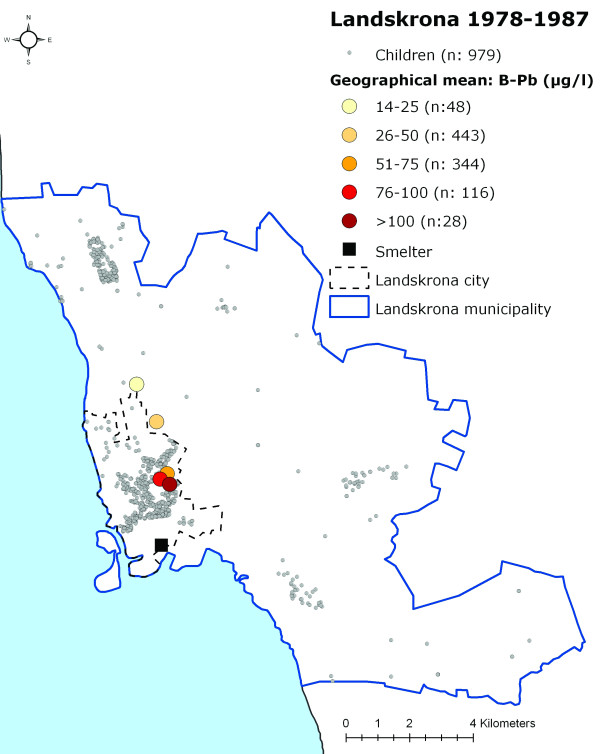
**The participating children's homes in Landskrona and the data categorised according to B-Pb level 1978–1987**. The location of the participating children's homes in Landskrona and the location of the geographical mean for the children in different B-Pb categories during 1978–1987.

**Figure 5:**
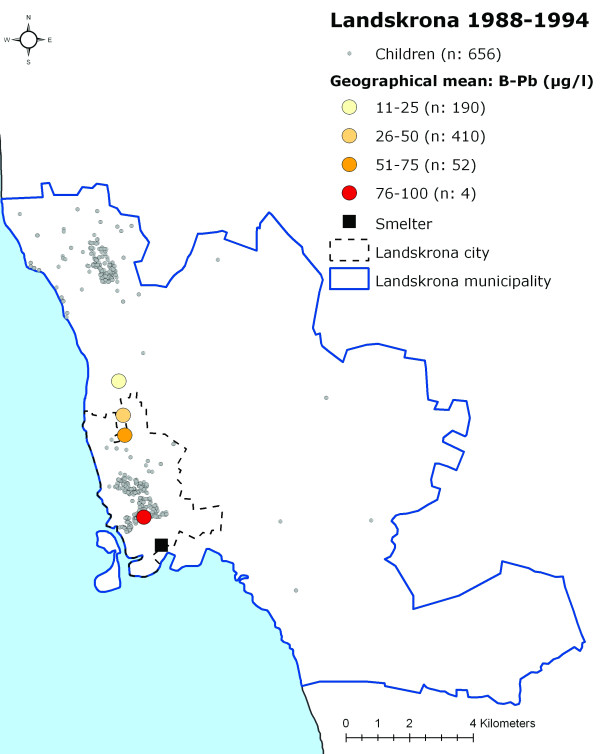
**The participating children's homes in Landskrona and the data categorised according to B-Pb level 1988–1994**. The location of the participating children's homes in Landskrona and the location of the geographical mean for the children in different B-Pb categories during 1988–1994 (one child had a B-Pb level above >100 μg/l (120 μg/l), and this child and category were therefore excluded from the analysis).

**Figure 6:**
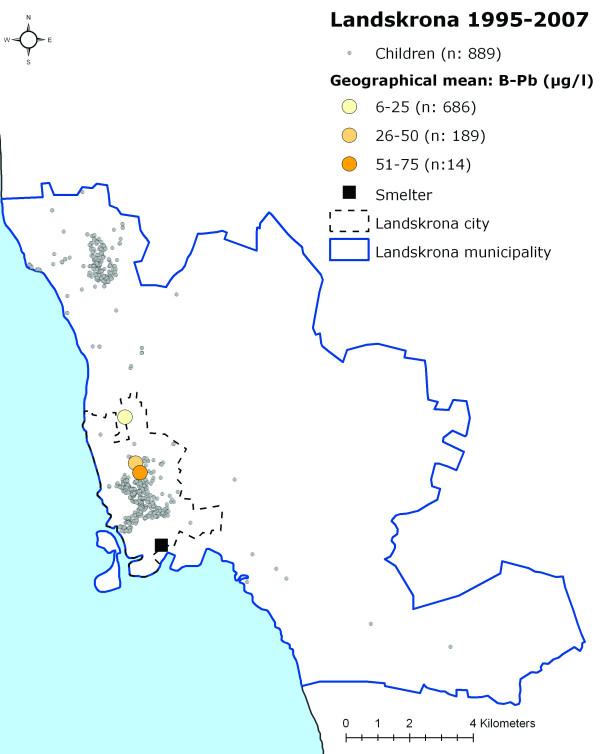
**The participating children's homes in Landskrona and the data categorised according to B-Pb level 1995–2007**. The location of the participating children's homes in Landskrona and the location of the geographical mean for the children in different B-Pb categories during 1995–2007 (one child had a B-Pb level above >75 μg/l (80 μg/l), and this child and category were therefore excluded from the analysis).

When grouping the children into categories according to their measured B-Pb levels (0–25 μg/l, 26–50 μg/l, 51–75 μg/l, 76–100 μg/l and >100 μg/l) and calculating a geographical mean for each of these categories, a distinct geographical pattern appeared, with the highest B-Pb levels closest to the smelter, thereafter decreasing with increasing distance from the smelter (Figures 4, 5 and 6).

**Figure 7:**
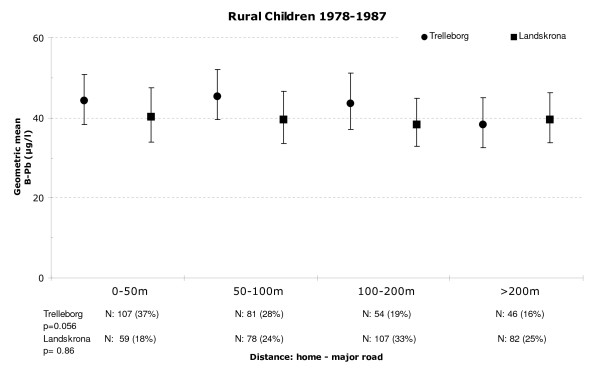
**Geometric mean blood lead levels of rural children in Trelleborg and Landskrona**. Geometric mean blood lead levels of rural children in Trelleborg and Landskrona from 1978–1987, as a function of the distance between their homes and major roads. Whisker represents the 95% confidence interval.

In order to quantify the effect of lead exposure resulting from traffic, we first analysed the effects of rural children's proximity to major roads. By excluding the urban children in Landskrona, we avoided including the near-smelter effect. We found no statistically significant association between the B-Pb levels of the rural children in Landskrona and their proximity to major roads during any of the time periods; 1978–1987: p = 0.9 (Figure 7), 1988–1994: p = 0.4 and 1995–2007: p = 0.12. For the rural children in Trelleborg, on the other hand, a weak effect was detected during the earliest time period, i.e., 1978–1987 (p = 0.056; Figure 7). However, a significant difference in B-Pb levels was found between urban and rural children in Trelleborg (urban geometric mean: 49.5 μg/l; rural geometric mean: 46.1 μg/l) during the earliest period (p = 0.014). The same significant difference between urban and rural children was also found in Landskrona during the same period. To ensure that there was no interaction with the near-smelter effect, children living within 2, 2.5 and 3 km, respectively, from the smelter were excluded from the urban category in succession (p < 0.001; p < 0.001 and p = 0.009).

## Discussion

We have studied the geographical patterns of children's B-Pb levels within different periods (1978–1987, 1988–1994 and 1995–2007). The geographical pattern in Landskrona, with respect to proximity to the lead smelter persisted throughout all periods. In fact, an analogous geographical gradient was seen when geocoding the children according to their school location only, without considering their home location (data not shown). One reason for the persistence near-smelter effect over the years could be that the lead emissions from the smelter in Landskrona have been, and still are, significant enough to cause elevated B-Pb levels in children living in its vicinity. The local authorities in Landskrona have made regular measurements of the heavy metals contents in the air in and around the industrial area close to the smelter since 1988 by measuring the dry deposition (fall-out) from the air. According to these measurements, dust deposition is characterized by a high amount of lead, especially in the industrial area around the lead smelter (in 2007: 200 mg/m2 per year, Jonsson, 2008). During the past twenty years, this deposition of lead from the smelter has not decreased but has remained between 200 and 300 mg/m2 per year, with a small tendency towards an increase during recent years [14]. The deposition of lead in Landskrona in 2006 has been found to be more than 50 times higher than in some other Scandinavian cities: Malmo and Stockholm, in Sweden, and Mo, in Norway [14], and measurements of lead in mosses show that the levels in Landskrona (especially in the city) were three times higher than the average in Scania in 2005 [15].

Although lead emissions from the smelter have been reasonably stable during the past twenty years, they are noticeably lower than during the early 1970s, when the lead deposition levels from the air in the vicinity of the smelter averaged roughly 600 mg/m2 per year [16]. For this reason, another possible explanation of the exposure pattern seen in Figures 4, 5 and 6 may be that the lead accumulated in soil in the surroundings of the smelter is still high enough to influence the observed near-smelter gradient of B-Pb levels. It is possible that the effect of industrial exposure decreases slowly in people living in the vicinity of a lead source, despite the fact that the emission decreased many years ago. The implications of such long-lasting effects of industrial emission should be taken seriously, especially since several other exposure studies on children living in the vicinity of lead sources have shown higher B-Pb levels than those of the children in our study [5-8,17-19].

The results obtained from the analysis of the proximity of children's homes to the closest major road in rural areas of Trelleborg indicate that proximity to major roads, and thus exposure to traffic-generated lead, might have had a slight effect on B-Pb levels during the early period, 1978–1987. This is in accordance with other studies of lead exposure in areas where leaded petrol was in use [1]. However, this association was weak, and we found no such evidence for the rural children living in Landskrona. We did not include data from urban children in the overall analyses of proximity to major roads, as it would have been difficult to compensate for emissions from the urban traffic network and multiple major roads in their vicinity, as already mentioned, we wanted to avoid any possible effects of the smelter in Landskrona. Due to the fact that we analysed the distance to the closest major road in the children's vicinity we might have underestimated the effect from exposure through traffic since the road influence for children living on the same distance to several major roads was not measured. Nevertheless, during the early period with high levels of lead in petrol (1978–1987), we observed that urban children in both Landskrona and Trelleborg had significantly higher B-Pb levels than the rural children, and that this difference persisted in Landskrona when children living within 2, 2.5 and 3 km of the smelter were successively removed from the analysis. Although the reason for this could be caused by distinction in food habits among urban and rural children the most probable explanation is that it is due to different exposure to traffic exhaust.

Although the total Swedish emission of lead has decreased, children may still be exposed to lead in air from other regions or countries in the vicinity [20]. However, it seems unlikely that such lead exposure would influence geographical patterns locally.

In exposure studies it is always hard to account for all the possible ways of exposure. Younger children (preschool) might for example be exposed to vast quantities of lead from contaminated soil by hand-to-mouth activity due to their tendency to put things in their mouth. However, in our study the children were predominantly aged between 8 and 10 years of age (only 77 children in the study were younger than 8 years and this age span was only a part of the study during 1986). We therefore consider the children to be beyond the "hand-to-mouth" age and thus this exposure factor should be of no major concern for our study. Another important exposure factor is parental occupation, i.e. if the parents are exposed to lead in their occupation and thereby might bring home contaminated clothes and pollute the child's home environment. Information about parental work exposure to lead and their children's B-Pb have been analysed in previous studies [3,21]. In short; only a few children had parents that worked within lead related industry and the results from the analyses showed no evidence for parental work exposure to be influential in neither study which is probably due to the restrictive work policies that were introduced in the beginning of the 1970. Consequently we consider the impact of parental work exposure of minor concern. Also no children that were part of our study worked part-time at the smelter.

In our study design it was impossible to account for individual exposure that might originate from individual behaviour and sources such as food intake, consumption of homeopathic substances or the use of leaded household utensils. It is also possible that children recently immigrated to Sweden could have elevated B-Pb levels that were caused by lead exposure in their previous country rather than that of their present. Unfortunately we lacked information about the date of immigration and were therefore unable to adjust for this. There is also a risk that the children could differ in terms of susceptibility to lead exposure (however, no such data are available).

We have no reason to believe that the selection bias for the children participating in this study should be substantial due to the homogenous participation rate in all the schools during the whole study period. Although we miss information about the children that did not participate in the study there is no reason to believe that these should be geographically clustered or spatially distributed, along with differences in individual susceptibility, to an extent that would affect our results.

The geometrical means of the different periods can not be expected to be directly comparable because: (i) parents' smoking habits could not be adjusted for in the early period, 1978–1987, and (ii) the covariate effects and data distributions differ somewhat across the periods. However, the results remained when the periods were analysed separately with and without smoking as a fixed-effect factor and analyses based on combined periods: 1988–1994 and 1995–2007 (meaning that the estimated geometric means are directly comparable across these two periods, with adjustment for parental smoking) and 1978–1987, 1988–1994 and 1995–2007 (meaning that the estimated geometric means are directly comparable across the three periods, without adjustment for parental smoking) showed that the incomparability between periods is not large (GMs did not differ more than 7%)", except fort the period 1988–1994 in Trelleborg (GMs differed between 13–17%, i.e. corresponding to an increase in B-Pb levels with 3.3–4.4 μg/l) which is due to mentioned differences in data distribution and covariates. Importantly, we found no evidence for a geographical pattern, regardless of data analysis.

Another limitation was that we obtained the location of the children's homes (i.e. the coordinates) from the Regional Population Register, which is based on the location of each citizen at the end of each year. If the children had changed address late in the year, the exposure estimate for that year would have been based on the new address rather than the old one. The coordinates were also obtained for the centre of their residence which, for large estates, e.g. farms, could lead to an erroneous location. However, we do not believe these approximations would have had any significant effects on the results.

As reported in detail in earlier studies on the same children, the quality of the B-Pb determinations was good [2-4]. The present B-Pb levels are low in comparison to those encountered in many other countries. However, the concentrations at the beginning of the study period were in the range now strongly suspected to induce effects on the central nervous system in infants.

Spatial analysis was a valuable tool in this study, making it possible to analyse the influence of sources of lead exposure with high accuracy. The inclusion of geographical analyses together with high-resolution census data made it possible to analyse different exposure pathways. The possibility of linking each child with the actual coordinates of their home and school in each year of interest allowed us to determine their proximity to lead sources and thus more accurately model their possible exposure.

The overall decrease in B-Pb levels that has been observed in Southern Sweden during the past thirty years is substantial [2-4,21] although this is not definite proof it is highly likely that this is due to the reduction and later prohibition of lead in petrol [2-4], and is in accordance with the results of studies in other countries [18,22-24]. However, dust deposition measurements shows that the lead emissions from the smelter in Landskrona have not decreased, but remained rather stable since 1988, and there is a tendency towards an increase in the emission levels in the industrial area. Present emissions and the likelihood that previous years emissions might have caused an accumulation of lead in the soil nearby the smelter could be the reason to why there still is a connection between the children's proximity to the smelter and their B-Pb levels.

## Conclusion

The geographical analysis revealed that, although the levels of lead in children's blood have decreased considerably since 1978, proximity to the lead smelter has continued to affect their B-Pb level. This is alarming since it might imply that, living or working in the vicinity of a former lead source could pose a threat year after reduction of the emission. The implications of such long-lasting effects must be taken seriously. Also, our analysis has revealed that urban children exposed to lead from traffic were only affected during the early period, when there were considerable amounts of lead in petrol, and that the prohibition of lead in petrol in later years led to reduced levels of lead in the blood of urban children.

## Competing interests

The authors declare that they have no competing interests.

## Authors' contributions

ES carried out the geographical analysis, participated in the design of the study and drafted the manuscript. TL carried out the data mining and participated in the design of the study. AO performed the statistical analysis. SS conceived of the study, and participated in the design of the study. US conceived of the study, and participated in its design and coordination and helped to draft the manuscript. All authors read and approved the final manuscript.

## Pre-publication history

The pre-publication history for this paper can be accessed here:


